# Mental Distress Among Female Individuals of Reproductive Age and Reported Barriers to Legal Abortion Following the US Supreme Court Decision to Overturn *Roe v Wade*

**DOI:** 10.1001/jamanetworkopen.2023.4509

**Published:** 2023-03-23

**Authors:** Dhaval Dave, Wei Fu, Muzhe Yang

**Affiliations:** 1 Department of Economics, Bentley University, Waltham, Massachusetts; 2 Department of Pathology and Laboratory Medicine, Perelman School of Medicine, University of Pennsylvania, Philadelphia; 3 Leonard and Davis Institute of Health Economics, University of Pennsylvania, Philadelphia; 4 Department of Economics, Lehigh University, Bethlehem, Pennsylvania

## Abstract

This case-control study investigates the association of the US Supreme Court decision to overturn *Roe v Wade* with mental distress among female individuals of reproductive age.

## Introduction

The Supreme Court of the US (SCOTUS) ruling in *Dobbs v Jackson Women’s Health Organization* (hereafter, the *SCOTUS decision*), issued on June 24, 2022, overturned *Roe v Wade*, allowing states to set their own abortion laws, including outright bans. The American Psychological Association expressed alarm that eliminating the constitutional right to abortion would harm women’s mental health and exacerbate the ongoing mental health crisis in the US. We assessed whether the SCOTUS decision was associated with mental distress among female individuals of reproductive age and how this association varied by barrier to legal abortion.

## Methods

Analyses of secondary, deidentified data in this case-control study were determined to be exempt from institutional review board review by Lehigh University. This study followed the Strengthening the Reporting of Observational Studies in Epidemiology (STROBE) reporting guideline.

We used individual-level data from the Census Bureau Household Pulse Survey, January 26 to September 28, 2022. This survey has been used to study mental health^1^ and contains information on respondent sociodemographics and residential state, to which we matched information on the status of state abortion bans from the Guttmacher Institute and travel distance to the nearest abortion clinic from Myers et al.^2^

We used a difference-in-differences model comparing changes before and after leak of the SCOTUS draft opinion and after the SCOTUS decision in mental distress among female individuals living in states where abortions had been or would likely be banned vs states where abortion rights continued to be protected, while accounting for confounders. We conducted event-time analyses to assess dynamics in the association between losing unrestricted access to abortion and mental distress. Furthermore, we examined whether this association varied by barrier to legal abortion as proxied by state-level changes in travel distances to the nearest abortion clinics. Estimations used ordinary least squares following previous studies, such as Raifman et al.^3^ Statistical tests were 2-tailed *t* tests using the 95% significance level. Our conclusions remained unchanged when using logistic regressions. See eMethods in Supplement 1 for details.

## Results

For 83 313 female individuals of reproductive age (aged 18-44 years; mean [SD] age, 32.9 [6.9] years; 13.2% Black and 73.5% White; 20.6% Hispanic) residing in states restricting abortion rights post-SCOTUS decision, we found a statistically significant higher prevalence of mental distress after the ruling (increase in prevalence, 0.042; 95% CI, 0.009-0.075], a 10.0% increase vs the preperiod proportion of 0.418) and that there was an interaction between changes in barriers to legal abortion and the association between the SCOTUS decision and mental distress (increase in prevalence, 0.012; 95% CI, 0.005-0.019). Among 152 402 female individuals older than reproductive age (aged 45-75 years; mean [SD] age, 59.9 [8.6] years; 12.5% Black and 78.3% White; 12.7% Hispanic), there were no such associations (Table).

**Table.** Association Between Losing the Constitutional Right to Abortion and Mental Distress

Factor	Mental distress prevalence (95% CI)	
	Female individuals aged 18-44 y	Female individuals aged 45-75 y
Abortion ban status × postdescision^a^^,^^b^	0.042 (0.009 to 0.075)	0.006 (−0.010 to 0.022)
Observations, No.	83 313	152 402
Clusters, No.	49	49
Preperiod proportion with mental distress	0.418	0.266
Change in distance to nearest abortion clinic × postdecision^c^	0.012 (0.005 to 0.019)	0.002 (−0.002 to 0.005)
Observations, No.	83 313	152 402
Clusters, No.	49	49
Preperiod proportion with mental distress	0.418	0.266
Control variables used in both analyses, status		
Individual demographics^d^	Yes	Yes
State-level time-varying characteristics^e^	Yes	Yes
State fixed effect	Yes	Yes
HPS wave fixed effect	Yes	Yes

Abbreviation: HPS, Household Pulse Survey.

^a^ Estimations are based on a revised difference-in-differences model (Supplement 1); use a linear probability model in which the dependent variable is a binary, equal to 1 or 0 for having or not having mental distress in the past 2 weeks; are weighted by HPS survey weights; and use HPS from wave 42 (January 26 to February 7, 2022). CIs are computed using standard errors clustered at the state level. The variable postdescision is binary, equal to 1 for waves 46 (June 1-13, 2022) to 49 (September 14-28, 2022) and 0 for waves 42 (January 26 to February 7, 2022) to 45 (April 27 to May 9, 2022).

^b^ The abortion ban status is a binary variable (1: trigger ban, bans or restrictions will take effect, bans or restrictions likely vs 0: has not banned or protects abortion rights).

^c^ Change in distance to the nearest abortion clinic is a continuous, nonnegative variable from Myers et al,^2^ 2019 (Supplement 1).

^d^ Includes age, age squared; White race (1/0 [ie, yes or no]); Black race (1/0) Hispanic, Latino, or Spanish origin (1/0); educational attainment; marital status; income level; and occupation.

^e^ Includes mean of newly confirmed COVID-19 cases per 100 000 people in the past 30 days prior to each HPS wave, mean of COVID-19 vaccine series completed per 100 people in the past 30 days prior to each HPS wave, and the number of initial claims for regular unemployment insurance per 100 people in the 2019 labor force in the month prior to each HPS wave.

These findings were supported by event-time analyses (Figure). Associations of abortion restrictions (Figure A) and increased travel distance to the nearest abortion clinic (Figure B) with prevalence of mental distress for female individuals of reproductive age persisted, with increased coefficients over time. There was no statistically significant increase in prevalence of mental distress after the leak of the SCOTUS draft opinion until some time after the ruling.

**Figure.**
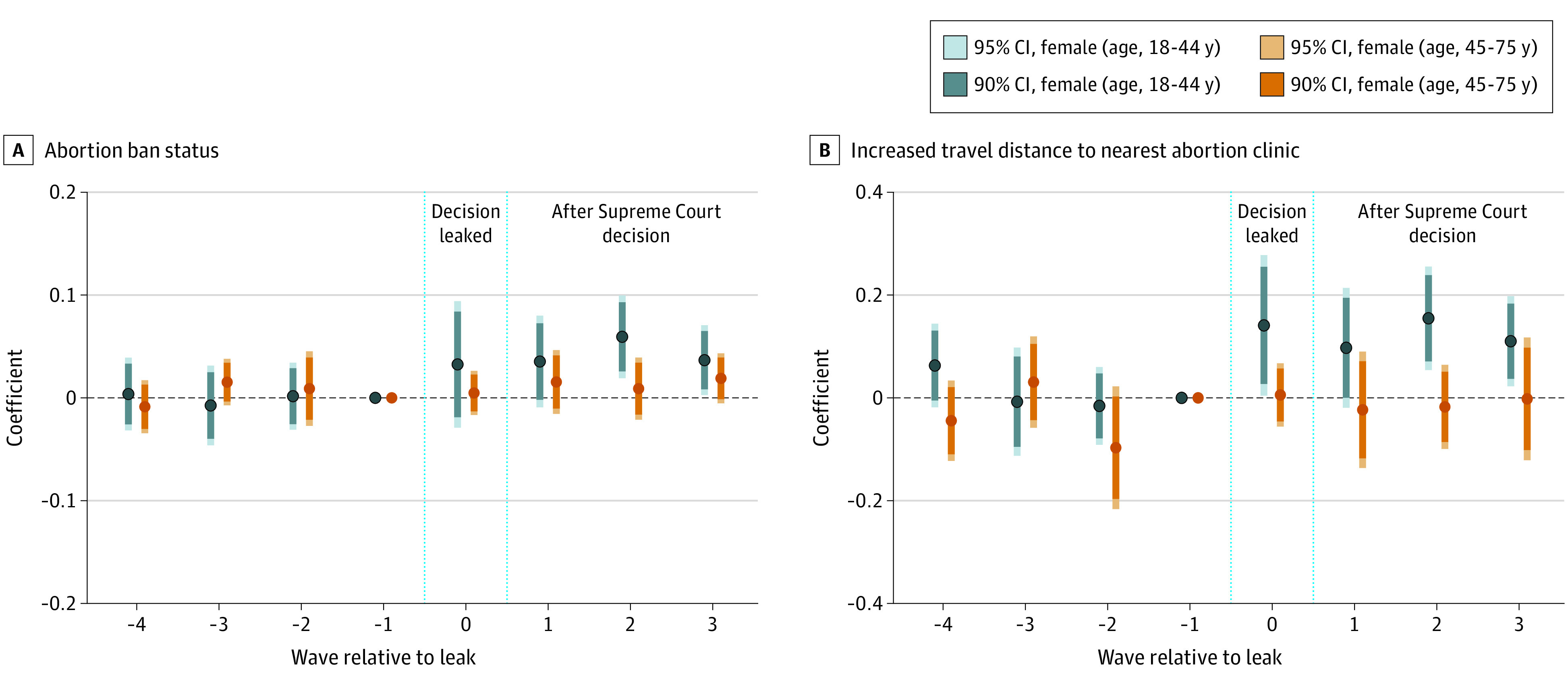
Changes Over Time in Mental Distress Associated With Abortion Ban Status and Increased Distance to Nearest Clinic

## Discussion

Losing the constitutional right to abortion can be associated with women’s reproductive health directly and indirectly via how future obstetricians-gynecologists would be trained.^4^ This case-control study found that for female individuals, the loss of abortion rights was associated with a 10% increase in prevalence of mental distress relative to the mean over the 3 months after the SCOTUS decision. Restricting legal abortion access may be associated with disproportionate outcomes among individuals of lower socioeconomic status and in medically underserved areas, who may experience greater economic and mental health burdens of having unwanted pregnancies due to increased travel costs of obtaining abortions. Our study suggests that mental health outcomes associated with restricting abortion access may extend broadly, beyond female individuals who have been denied an abortion^5^ to female individuals of reproductive age. This study’s limitations include unmeasured confounders, such as the value individuals placed on safe and legal access to abortion. Individuals placing a high or low value could have increased or reduced mental distress after the SCOTUS decision. Our study was unable to analyze this heterogeneity.

## Notes

Supplement 1.
eMethods.

eReferences.

Supplement 2.
Data Sharing Statement
