# Supplementary material for: Mental Distress Among Female Individuals of Reproductive Age and Reported Barriers to Legal Abortion Following the US Supreme Court Decision to Overturn Roe v Wade
Source: JAMA Netw Open. 2023 Mar 23;6(3):e234509. doi: 10.1001/jamanetworkopen.2023.4509 (PMC10037155; doi:10.1001/jamanetworkopen.2023.4509)
Supplement: Supplement 1. — eMethods. eReferences. [file jamanetwopen-e234509-s001.pdf]

Supplemental Online Content

Dave D, Fu W, Yang M. Mental distress among female individuals of reproductive age and reported barriers to legal abortion following the US Supreme Court decision to overturn *Roe v Wade*. *JAMA Netw Open*. 2023;6(3):e234509. doi:10.1001/jamanetworkopen.2023.4509

eMethods.

eReferences.

This supplemental material has been provided by the authors to give readers additional information about their work.

© 2023 Dave D et al. *JAMA Network Open.*

eMethods

1. Data Sources

1.1. Main data source

Our main data source is the Household Pulse Survey (HPS). [1] The HPS was initiated by the U.S. Census Bureau, in collaboration with multiple federal agencies, with the purpose of tracking household experiences along social, economic, and health dimensions over the COVID-19 pandemic. The HPS collects repeated cross-sectional data, using an approximately two-weeks on, two-weeks off collection and dissemination approach. [2] The first wave of the HPS started in April 2020. By September 2022, the HPS had collected 49 waves of data.

In conjunction with the National Center for Health Statistics, the HPS collects data on respondents' mental health. Specifically, the HPS asks respondents to self-report their mental health using the following four questions:

- Q1: *"How often have you been bothered by feeling nervous, anxious, or on edge in the past two weeks?"*
- Q2: *"How often have you been bothered by not being able to stop or control worrying in the past two weeks?"*
- Q3: *"How often have you been bothered by having little interest or pleasure in doing things in the past two weeks?"*
- Q4: *"How often have you been bothered by feeling down, depressed, or hopeless in the past two weeks?"*

Answers to these questions (i.e., Q1 through Q4) are measured using ordinal scales: "not at all," "several days," "more than half the days," and "nearly every day." Here, we closely followed Agrawal et al. (2021), who also used the HPS data on the answers to these four questions to study trends in mental health. First, those ordinal scales were coded as 0 (for "not at all"), 1, 2 and 3, respectively. Next, the numeric scores on Q1 and Q2 were added to create a binary variable, which is equal to one if the total score is at least 3, and equal to zero otherwise. Agrawal et al. (2021) referred to this variable as generalized anxiety disorder (GAD). Similarly, the scores on Q3

---

[1] Data are downloadable from https://www.census.gov/programs-surveys/household-pulse-survey/datasets.html (accessed on October 12, 2022).

[2] For example, Wave 46 of the HPS was conducted from June 1, 2022 to June 13, 2022. The next wave, which is Wave 47, was conducted from June 29, 2022 to July 11, 2022.

© 2023 Dave D et al. *JAMA Network Open.*

and Q4 were added to create another binary variable, which is equal to one if the total score is at least 3, and equal to zero otherwise. Agrawal et al. (2021) referred to this variable as major depressive disorder (MDD). For our study, we constructed a binary outcome variable, which we refer to as mental distress, by combining GAD and MDD: the mental distress variable is equal to one if either GAD or MDD is equal to one, and equal to zero otherwise.

From the HPS we also obtained several variables regarding respondents' characteristics, which we used as control variables in our analysis. These variables, all measured at the individual level, are: age, white (1/0), black (1/0), Hispanic origin (1/0), five education levels (less than high school degree, high school degree or equivalent, some college or associate's degree, bachelor's degree, and graduate degree), marital status (married or not, and an indicator for missing values), nine income levels (under $25,000; $25,000 to $34,999; $35,000 to $49,999; $50,000 to $74,999; $75,000 to $99,999; $100,000 to $149,999; $150,000 to $199,999; $200,000 above; and an indicator for missing values), and seven occupation categories (unemployed, government, private company, non-profit organization including tax-exempt and charitable organizations, self-employed, working in a family business, and an indicator for missing values). Race and ethnicity were self-reported. Categories for race were Asian, Black, White, and other race alone or in combination; respondents could choose yes or no for Hispanic, Latino, or Spanish origin. We controlled for race and ethnicity because they are likely associated with mental health. In our analyses, we used 3 dummy variables (White, Black, and Hispanic, Latino, or Spanish origin); the comparison group included individuals who were not White, Black or of Hispanic, Latino, or Spanish origin.

In addition, the HPS has information on which state the respondent lives in. We used this information to link the HPS data with other data, which are described in the next section.

1.2. Other data sources

We obtained information from the Guttmacher Institute on individual states' status regarding banning or restricting abortions in the post-Roe v. Wade period. Using this information, we assigned each state, where the HPS respondent lives, into one of these four categories: "trigger ban," "bans or restrictions will take effect," "bans or restrictions likely," and "hasn't banned, or

© 2023 Dave D et al. *JAMA Network Open.*

protects."[3] Below is the table showing these categories and the associated states.[4] Based on this table we created a binary variable, which is equal to one for categories (A), (B) and (C); and equal to zero for category (D).

Table: Abortion Ban Status

Categories	States
(A) Trigger ban (12)	Arkansas, Idaho, Kentucky, Louisiana, Mississippi, North Dakota, Oklahoma, South Dakota, Tennessee, Texas, Utah, Wyoming
(B) Bans or restrictions will take effect (10)	Alabama, Arizona, Georgia, Iowa, Michigan, Missouri, Ohio, South Carolina, West Virginia, Wisconsin
(C) Bans or restrictions likely (4)	Florida, Indiana, Montana, Nebraska
(D) Hasn't banned, or protects abortion right (23)	California, Colorado, Connecticut, Delaware, District of Columbia, Illinois, Kansas, Maine, Maryland, Massachusetts, Minnesota, Nevada, New Hampshire, New Jersey, New Mexico, New York, North Carolina, Oregon, Pennsylvania, Rhode Island, Vermont, Virginia, Washington

Data source: Guttmacher Institute (https://www.guttmacher.org/).

In our study the variable on the change in the travel distance to the nearest abortion clinic is a state-level variable, which we obtained directly from Myers et al. (2019). In their study the travel distance to the nearest abortion clinic was first calculated for each county's population centroid for two cases, respectively: (a) abortion becomes illegal in eight states with trigger bans; (b) abortion becomes illegal in 13 additional states classified as being at high risk of outlawing abortions under most circumstances. In the next step, they aggregated the variable on the travel distance to the nearest abortion clinic from the county level to the state level. In our study we calculated the state-level average change in the travel distance to the nearest abortion clinic using (b) minus (a).

[3] The trigger ban means banning abortion as soon as Roe v. Wade is overturned by the Supreme Court. See the Center of Reproductive Rights for more details: https://reproductiverights.org/maps/abortion-laws-by-state/.

[4] In the table there are 48 states (i.e., the 48 contiguous states) plus the District of Columbia. In our study we dropped Alaska and Hawaii. This is because the travel distance variable used in our study, which we obtained from Myers et al. (2019), was constructed using only the 48 contiguous states and the District of Columbia.

© 2023 Dave D et al. *JAMA Network Open.*

In the data we obtained from Myers et al. (2019) all state-level average changes (in the travel distance to the nearest abortion clinic) have non-negative values, and the mean of these average changes is 138 miles. An increase in the travel distance is consistent with a decrease in the number of abortion clinics due to increased likelihood of outlawing abortion rights in more states. We merged this state-level variable into the HPS data, using the HPS's state identifier. Because the HPS data do not have identifiers for geographic areas smaller than states, we are only able to obtain a raw measure (which is at the state level) on the changes in travel distances to the closest abortion clinics. Because of this limitation, we interpret the numeric values of this variable, which are all non-negative, as an indication of increased barriers to abortion access, as opposed to the exact change in the travel distance to the closest abortion clinic.

Other control variables measured at the state level used in our study include the average of newly confirmed COVID-19 cases per 100,000 people in the past 30 days prior to each HPS wave, the average of the COVID-19 vaccine series completed per 100 people in the past 30 days prior to each HPS wave, [5] and the number of initial claims for regular unemployment insurance (UI) per 100 people in the 2019 labor force in the month prior to each HPS wave. [6] These data were obtained from Opportunity Insights. [7]

2. Regression Models

We set up our regression models using a difference-in-differences (DID) framework (see Dimick and Ryan, 2014), which is a research design often used in the evaluation of a policy change between two groups in two periods: one group (the treatment group) experienced the policy change in the second period (i.e., the post-period) but not in the first period (i.e., the pre-period), while the

[5] For example, for HPS Wave 42, which started on January 26, 2022, we merged the COVID data measured up to January 25, 2022, to this wave.

[6] More precisely, for an HPS survey wave that started in the first half of a month, we merged the UI claims data collected in the month before the HPS survey wave's month. For example, for Wave 43 (which started on March 2, 2022), we merged the UI claims data collected in February 2022. For an HPS survey wave that started in the second half of a month, we merged the UI claims data collected in the same month of the HPS survey wave. For example, for Wave 42 (which started on January 26, 2022), we merged the UI claims data collected in January 2022.

[7] For details, see https://opportunityinsights.org/.

© 2023 Dave D et al. *JAMA Network Open.*

other group (the comparison group) did not experience (or was unaffected by) the policy change in either period.

The HPS is ideal for our study because it collected data on respondents' mental health (and demographic characteristics) just before and after the leak of a draft opinion of the Supreme Court's decision to overturn Roe v. Wade, which happened on May 2, 2022. Within this short time frame, changes in respondents' mental health are plausibly associated only with the leaked decision. Furthermore, there are multiple waves of the HPS data in the post-leak period. These data allow us to evaluate whether changes in respondents' mental health are also associated with the actual decision of overturning Roe v. Wade, which happened on June 24, 2022, as well as how that association changed over time in the post-decision period.

According to the DID framework just described, we used data from Wave 42 (January 26–February 7 of 2022) through Wave 49 (September 14–September 28 of 2022) of the HPS (the most recent wave completed at the time of our study). In our study we focus on female respondents, and we conduct our analyses for two groups separately: (1) female individuals aged 18–44, who are of reproductive age and therefore may have a need for abortion access currently or in the future; (2) female individuals aged 45–75, who are beyond reproductive age and therefore far less likely to have a need for abortion access. Since the Supreme Court's decision affects women's abortion rights, we expect women of reproductive age to be more affected by the Supreme Court's decision. For each female sample, we use two regression models described in the following sections.

Our estimations use linear probability models (LPM), following studies that use LPM with a DID framework and binary outcome variables (e.g., Raifman et al., 2021). All hypothesis tests for the key parameters of the regression models (described in the following two sections) are based on two-tailed t-tests, conducted at the 95% level of significance.

2.1. Regression Model I

We specify Regression Model I as follows:

$$Y_{ist} = \beta_0 + \beta_1 AbortionBan_s \times Post_t + \beta_2 X_{ist} + \beta_3 Z_{st} + \gamma_s + \theta_t + \epsilon_{ist} \qquad (1)$$

© 2023 Dave D et al. *JAMA Network Open.*

Here, Y_{ist} is a binary variable indicating the mental distress status of female i living in state s and interviewed in wave t of the HPS. $AbortionBan_s$ is a binary variable, equal to one if state s is in categories (A), (B) and (C) shown in the table; and equal to zero if state s is in category (D) shown in the table. $Post_t$ is a binary variable, equal to one if wave t is any wave between 46 (conducted during June 1–June 13, 2022) and 49 (conducted during September 14–September 28, 2022), and equal to zero if wave t is any wave between 42 (conducted during January 26–February 7, 2022) and 45 (conducted during April 27–May 9, 2022). The estimate of β_1 is the DID estimate, indicating an association between losing abortion rights and mental distress among female individuals, in the period after the leak of a draft opinion of the Supreme Court's decision to overturn Roe v. Wade.

In equation (1) X_{ist} is a vector of the individual's characteristics, including age, age squared, white (1/0), black (1/0), Hispanic origin (1/0), educational attainment, marital status, income levels, and occupations; Z_{st} is a vector of state-level time-varying variables, including the average of newly confirmed COVID-19 cases per 100,000 people in the past 30 days prior to each HPS wave, the average of the COVID-19 vaccine series completed per 100 people in the past 30 days prior to each HPS wave, and the number of initial claims for regular unemployment insurance per 100 people in the 2019 labor force in the month prior to each HPS wave.

In equation (1) we also include state fixed effects (γ_s) to control for state-level time-invariant characteristics, as well as HPS wave fixed effects (θ_t) to control for common time trends across all states at each HPS wave. Note that the main effects of $AbortionBan_s$ and $Post_t$ are subsumed in these fully saturated state and wave fixed effects. Standard errors are clustered at the state level. All analyses are weighted by the HPS survey weights.

One key assumption in DID is that the treatment group and control group on average have the same change in the outcome variable between the pre-period and the post-period in the absence of the treatment. This assumption, often called the "parallel trends" assumption, involves a counterfactual for the treatment group in the post-period, so there is no direct test for the validity of this assumption. Nonetheless, it is standard for researchers to use an event-time version of equation (1) to test whether there are parallel trends in the pre-period and this presence provides some plausibility of the parallel trends continuing into the post-period. The event-time version of equation (1) is described by equation (2) below:

© 2023 Dave D et al. *JAMA Network Open.*

$$Y_{ist} = \beta_0 + \sum_{j=42, j\neq 45}^{49} \alpha_t AbortionBan_s \times 1\{t = j\} + \beta_2 X_{ist} + \beta_3 Z_{st} + \gamma_s + \theta_t + \epsilon_{ist}. \quad (2)$$

In equation (2) each HPS interview wave (from wave 42 to wave 49) is represented by a binary variable, with wave 45 (which is the last wave in the pre-period) used as the reference wave. In equation (2) zero values of the coefficients α_t's of the pre-period (i.e., $t < 45$) would indicate the presence of parallel trends in mental distress in the pre-period between states with risk of outlawing abortion rights and states without. The coefficients α_t's of the post-period (i.e., $t > 45$) indicate how the association between outlawing abortion rights and mental distress changes wave by wave throughout the post-period relative to the reference wave. The other variables' definitions in equation (2) are the same as in equation (1).

2.2. Regression Model II

In this model we use the same specifications used in equations (1) and (2), except that we replace the binary variable $AbortionBan_s$ with a continuous variable $ChangeDistance_s$, which we obtained directly from Myers et al. (2019), with detailed explanations provided in the "other data sources" section. In our regression analysis we rescaled $ChangeDistance_s$ to be in 100-mile units. The two new equations are described below.

$$Y_{ist} = \beta_0 + \beta_1 ChangeDistance_s \times Post_t + \beta_2 X_{ist} + \beta_3 Z_{st} + \gamma_s + \theta_t + \epsilon_{ist} \quad (3)$$

In equation (3) the estimate of β_1 indicates a change in the association between abortion access hurdle, as reflected by the travel distance to the nearest abortion clinic, and mental distress among female individuals, between the pre-period and the post-period (i.e., the period after the Supreme Court's decision to overturn Roe v. Wade was leaked).

The event-time version of equation (3) is described below:

$$Y_{ist} = \beta_0 + \sum_{j=42, j\neq 45}^{49} \alpha_t ChangeDistance_s \times 1\{t = j\} + \beta_2 X_{ist} + \beta_3 Z_{st} + \gamma_s + \theta_t + \epsilon_{ist} \quad (4)$$

Coefficients α_t's in equation (4) provide suggestive evidence about how the association between abortion access hurdle and mental distress changes over time, relative to the reference

wave (which is the last wave in the pre-period). If mental health of women of reproductive age is affected by the restricted access to abortion, we would expect that association to become more salient in the post-period (relative to the reference wave), when Roe v. Wade was overturned. This implies that relative to the reference wave, α_t's of the pre-period (i.e., $t < 45$) would be zero, but α_t's of the post-period (i.e., $t > 45$) can become positive, indicating an increase in the association between abortion access hurdle and mental distress.

Our conclusion is unaltered whether we use the ordinary least squares (OLS) regression model or the logistic regression model for estimation of the above specifications. These two models measure the association (described earlier) differently: in the OLS regression model, a positive (or negative) association is represented by the coefficient on the interaction term being greater (or smaller) than zero (indicating a change in the probability); in contrast, in the logistic regression model, a positive (or negative) association is represented by the exponentiated coefficient on the interaction term being greater (or smaller) than one (indicating a change in the odds ratio). We reported the OLS results in the manuscript and the logistic results are available upon request. The two sets of results are consistent.

© 2023 Dave D et al. *JAMA Network Open.*

eReferences

Agrawal, V., J. H. Cantor, N. Sood and C. M. Whaley, 2021. The Impact of the COVID-19 Vaccine Distribution on Mental Health Outcomes. *National Bureau of Economic Research* Working Paper 29593.

Dimick, J. B. and A. M. Ryan (2014). "Methods for Evaluating Changes in Health Care Policy: The Difference-in-Differences Approach." *JAMA* 312 (22): 2401–2402.

Myers, C., R. Jones and U. Upadhyay (2019). "Predicted Changes in Abortion Access and Incidence in a Post-Roe World." *Contraception* 100 (5): 367–373.

Raifman, J., J. Bor and A. Venkataramani (2021). "Association between Receipt of Unemployment Insurance and Food Insecurity among People Who Lost Employment during the COVID-19 Pandemic in the United States." *JAMA Network Open* 4 (1): e2035884–e2035884.

© 2023 Dave D et al. *JAMA Network Open.*
