# Supplementary material for: Mental Distress Among Female Individuals of Reproductive Age and Reported Barriers to Legal Abortion Following the US Supreme Court Decision to Overturn Roe v Wade
Source: JAMA Netw Open. 2023 Mar 23;6(3):e234509. doi: 10.1001/jamanetworkopen.2023.4509 (PMC10037155; doi:10.1001/jamanetworkopen.2023.4509)
Supplement: Supplement 2. — Data Sharing Statement [file jamanetwopen-e234509-s002.pdf]

Data Sharing Statement

Dave. Mental Distress Among Female Individuals of Reproductive Age and Reported Barriers to Legal Abortion Following the US Supreme Court Decision to Overturn *Roe v Wade*. *JAMA Netw Open*. Published online March 23, 2023.
doi:10.1001/jamanetworkopen.2023.4509

Data
Data available: Yes
Data types: Deidentified participant data
How to access data: https://www.census.gov/programs-surveys/household-pulse-survey/datasets.html https://opportunityinsights.org/
When available: With publication

Supporting Documents
Document types: None

Additional Information
Who can access the data: anyone requesting the data
Types of analyses: for any purpose or for a specified purpose
Mechanisms of data availability: by e-mail

© 2023 Dave D et al. *JAMA Network Open*.
